# The novel combination of dual mTOR inhibitor AZD2014 and pan-PIM inhibitor AZD1208 inhibits growth in acute myeloid leukemia via HSF pathway suppression

**DOI:** 10.18632/oncotarget.6122

**Published:** 2015-10-14

**Authors:** Masako Harada, Juliana Benito, Shinichi Yamamoto, Surinder Kaur, Dirim Arslan, Santiago Ramirez, Rodrigo Jacamo, Leonidas Platanias, Hiromichi Matsushita, Tsutomu Fujimura, Saiko Kazuno, Kensuke Kojima, Yoko Tabe, Marina Konopleva

**Affiliations:** ^1^ Research Institute for Environmental and Gender Specific Medicine, Juntendo University of Medicine, Tokyo, Japan; ^2^ Department of Laboratory Medicine, Juntendo University of Medicine, Tokyo, Japan; ^3^ Section of Molecular Hematology and Therapy, Department of Leukemia, The University of Texas MD Anderson Cancer Center, Houston, Texas, USA; ^4^ Division of Hematology-Oncology, Robert H. Lurie Comprehensive Cancer Center, Northwestern University Medical School, Chicago, Illinois, USA; ^5^ Department of Laboratory Medicine, Tokai University School of Medicine, Kanagawa, Japan; ^6^ BioMedical Research Center, Juntendo University of Medicine, Tokyo, Japan; ^7^ Laboratory of Bioanalytical Chemistry, Tohoku Pharmaceutical University, Miyagi, Japan; ^8^ Hematology, Respiratory Medicine and Oncology, Department of Medicine, Saga University, Saga, Japan

**Keywords:** acute myeloid leukemia (AML), mTORC1/2 dual inhibitor, PIM inhibitor, heat shock factor (HSF)

## Abstract

Mammalian target of rapamycin (mTOR) signaling is a critical pathway in the biology of acute myeloid leukemia (AML). Proviral integration site for moloney murine leukemia virus (PIM) serine/threonine kinase signaling takes part in various pathways exerting tumorigenic properties. We hypothesized that the combination of a PIM kinase inhibitor with an mTOR inhibitor might have complementary growth-inhibitory effects against AML. The simultaneous inhibition of the PIM kinase by pan-PIM inhibitor AZD1208 and of mTOR by selective mTORC1/2 dual inhibitor AZD2014 exerted anticancer properties in AML cell lines and in cells derived from primary AML samples with or without supportive stromal cell co-culture, leading to suppressed proliferation and increased apoptosis. The combination of AZD1208 and AZD2014 rapidly activated AMPKα, a negative regulator of translation machinery through mTORC1/2 signaling in AML cells; profoundly inhibited AKT and 4EBP1 activation; and suppressed polysome formation. Inhibition of both mTOR and PIM counteracted induction of heat-shock family proteins, uncovering the master negative regulation of heat shock factor 1 (HSF1), the dominant transcription factor controlling cellular stress responses. The novel combination of the dual mTOR inhibitor and pan-PIM inhibitor synergistically inhibited AML growth by effectively reducing protein synthesis through heat shock factor pathway suppression.

## INTRODUCTION

Acute myeloid leukemia (AML), the clonal expansion of hematopoietic progenitors characterized by acquired somatic mutations, represents one of the most common types of leukemia in adults, though it affects people of all ages [1]. Activation mutation of upstream regulators such as Fms-like tyrosine kinase 3 (*FLT3*) and *KIT* is a frequent event in AML pathogenesis, regulating various downstream pathways such as Ras/Raf/MEK/ERK, PI3K/PTEN/AKT/mTOR, and Jak/STAT and resulting in leukemogenesis [2]. However, targeted therapy in AML has achieved only partial success, and the mechanisms contributing to resistance are yet to be fully discovered.

Activated *FLT*3 signaling induces expression of the *STAT5* gene, which subsequently induces expression of proviral integration site for moloney murine leukemia virus 1 (PIM1) in AML [3]. *FLT*3 is the most frequently mutated gene in AML cells, and approximately 30% of AML patients harbor constitutively activating internal tandem duplication (ITD) mutations known to be associated with poor prognosis [2, 4-6].

PI3K/AKT/mTOR is an evolutionarily conserved pathway that plays an important role in regulating cell growth and proliferation. Mammalian target of rapamycin (mTOR) has been an attractive target for treatment of various types of tumors for years, but the efficacy and clinical activity of the first-generation mTOR inhibitors, which selectively target mTORC1, have been disappointing [4, 5]. Inability to inhibit mTORC2 is one of the potential key factors in rapamycin resistance, in which S6K1 and TSC1/2 are involved in a negative-feedback loop to regulate mTORC2 levels, resulting in cell survival and metabolic regulation [6].

AZD2014 is a second-generation dual mTORC1/2 inhibitor that has entered preclinical and clinical trials, showing highly specific activity against mTORC1/2 and thereby efficiently blocking the AKT/mTOR signal transduction pathway [5]. AZD2014 effectively inhibited mTORC1 targets phospho-(p-)S6K (Thr-389), p-4EBP1 (Thr-37/46), and mTORC2 target pAkt (Ser473), causing effective inhibition of the mTOR pathway without negative-feedback induction of mTORC2 in different types of tumor cells [7].

PIM proteins are considered “weak” oncoproteins because they require an accompanying oncoprotein to exert their tumorigenic properties. They are overexpressed in a broad range of tumors, including both hematological malignancies [8] and solid tumors [9]. The PIM protein family consists of 3 members (PIM1, 2, and 3) with tissue-specific expression distribution and overlapping functions; PIM1 and PIM2 are enriched in the hematopoietic system [10]. There have been several reports on the association of PIM1 with chemotherapy or radiotherapy resistance in various tumor types [11-15]. Though the PIM kinases are attractive pharmaceutical targets, targeting one member leads to development of resistance via the compensatory functions of the other members of the family because of the high degree of homology among them [16]. The highly specific pan-PIM inhibitor AZD1208 abolishes the compensatory effect from the other PIM family members and has been shown to have antitumor efficacy in both AML cell lines and primary samples [17]. PIM kinase signaling takes part in various pathways to define cell fate, including senescence, cell cycle regulation, apoptosis, metabolism, invasion, and metastasis; thus the combination of a PIM kinase inhibitor with an mTOR inhibitor is expected to offer greater antitumor effects in AML than either inhibitor alone [18]. Furthermore, PIM and AKT kinase inhibitors show synergistic cytotoxicity in AML associated with mTOR and MCL1 pathway repression [19].

Therefore, we hypothesized that these inhibitors used in combination might have complementary growth-inhibitory effects against AML, although no preclinical investigation of a regimen combining mTOR and PIM inhibitors against AML subtypes has been published. In this study, we used *in vitro* screening to identify the efficacy of a novel drug combination, pan-PIM inhibitor AZD1208 and dual-mTORC1/2 inhibitor AZD2014, and demonstrated that the combination of AZD2014 and AZD1208 synergistically inhibited growth of AML regardless of *FLT*3 mutation status through impaired polysome assembly and induction of apoptosis.

## RESULTS

### Expression of PIM1 kinase in AML cells

**Figure 1:**
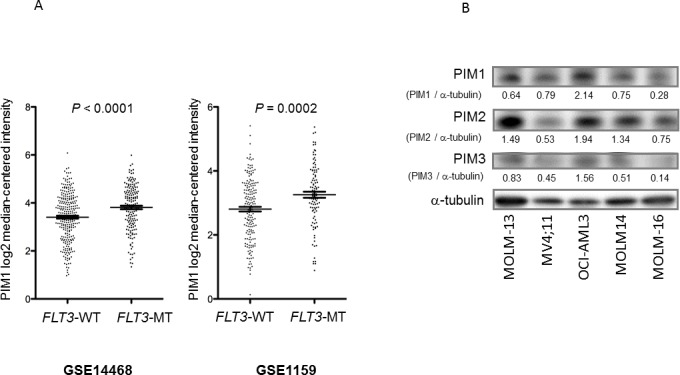
FLT3 mutations associated with high levels of *PIM1* mRNA **A.** *PIM1* mRNA levels were analyzed in AML patient samples with *FLT*3 wild-type (WT) or *FLT*3 mutant (MT) from 2 studies (GSE14468 and GSE1159) by the Oncomine Platform. Error bars indicate SEM. **B.** PIM1, PIM2, and PIM3 protein levels in AML cell lines with *FLT3*-WT (OCI-AML3, MOLM-16) or with *FLT3*-ITD (MV4;11, MOLM-13, MOLM-14) were detected by Western blotting. The intensities compared to those of α-tubulin levels after background subtraction were obtained using ImageJ software.

It has been reported that *FLT3*-ITD activation mutation leads to upregulation of PIM1 [13]. To examine whether *FLT*3 mutations are associated with high PIM1 expression levels in AML, we first determined the *PIM1* mRNA expression levels in AML patient samples from 2 large studies (GSE14468 and GSE1159) using the Oncomine Platform (Life Technologies, Ann Arbor, MI). These analyses showed greater expression of *PIM1* mRNA in the *FLT*3-mutant (*FLT3-*MT) samples than in the *FLT*3 wild-type (*FLT3*-WT) AML samples in both the GSE14468 series (*P* < 0.001; 168 *FLT3-*MT samples versus 276 *FLT3-*WT samples) and the GSE1159 series (*P* = 0.002; 106 *FLT3-*MT samples versus 178 *FLT3-*WT samples) (Figure 1A). Immunoblot analysis to determine the protein expression levels of PIM1 along with PIM2 and PIM3 of AML cell lines with known *FLT*3-mutation status (*FLT3*-WT; OCI-AML3, MOLM-16, *FLT3-*ITD; MV4;11, MOLM-13, MOLM-14) revealed no association of PIM expression and *FLT3* mutation status (Figure 1B). The PIM1 protein levels of 13 primary AML samples (9 with *FLT3*-WT and 4 with *FLT3-*ITD) further demonstrated no distinct relevance to *FLT*3-mutation status (Figure S1). These results suggest that *FLT3* mutations might be associated with high levels of *PIM1* mRNA but not protein expression in AML.

### Individual and synergistic effects of AZD1208 and AZD2014 in AML cells

**Figure 2:**
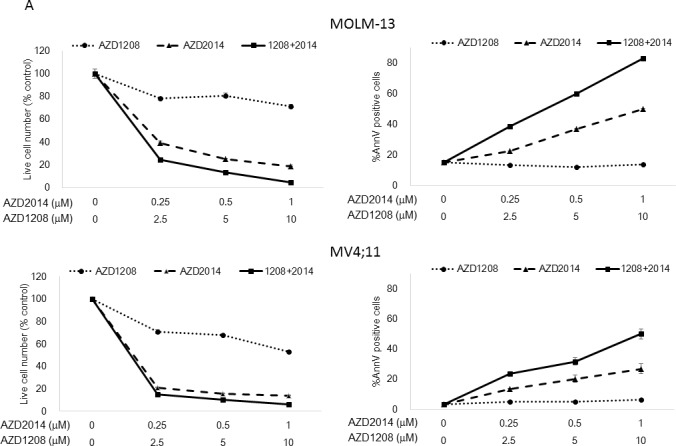

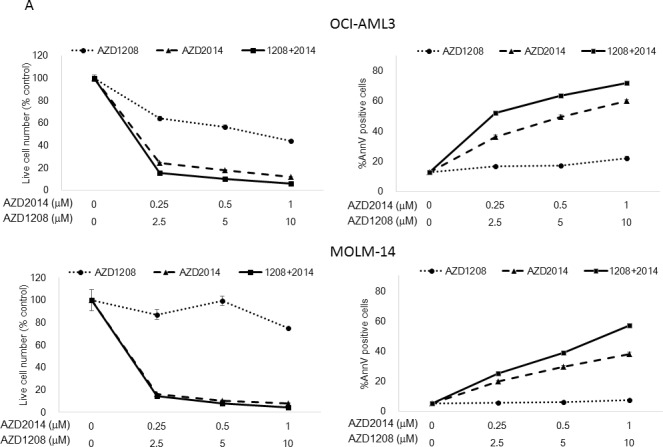

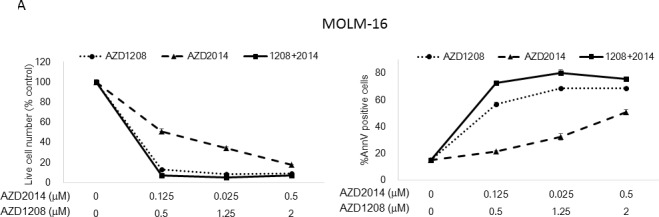

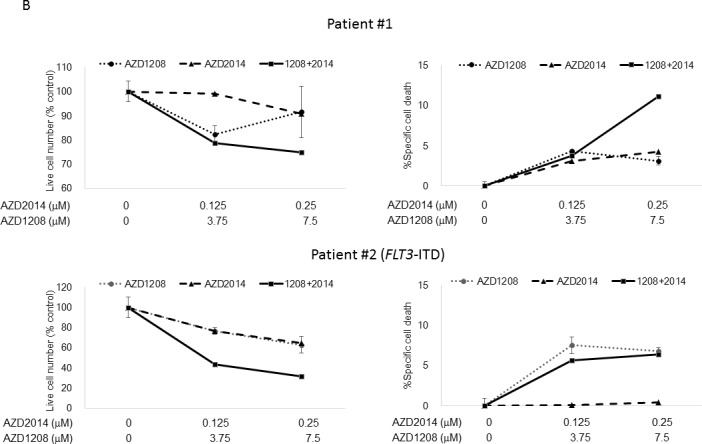

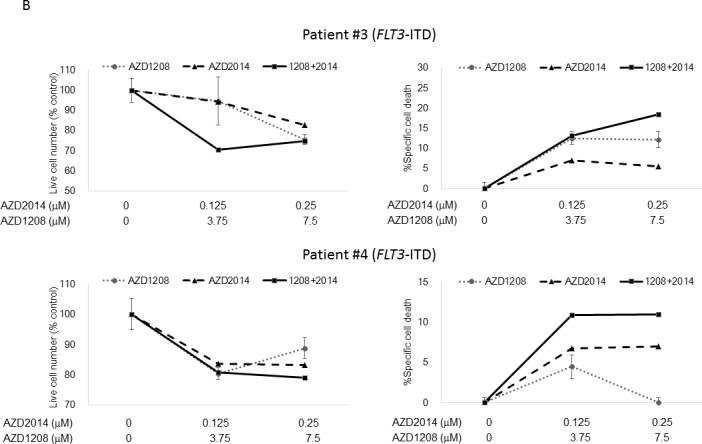

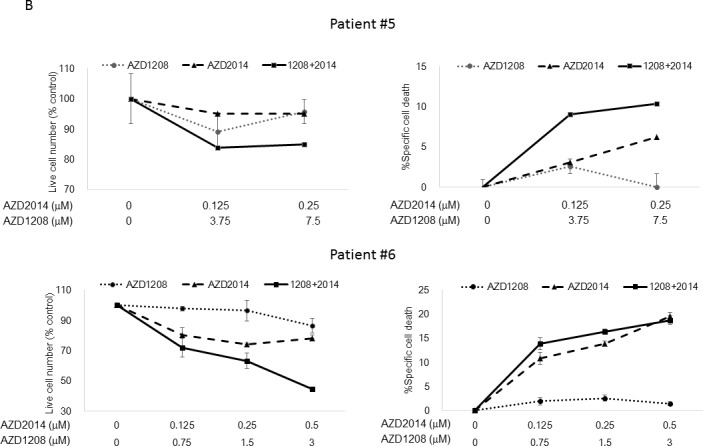

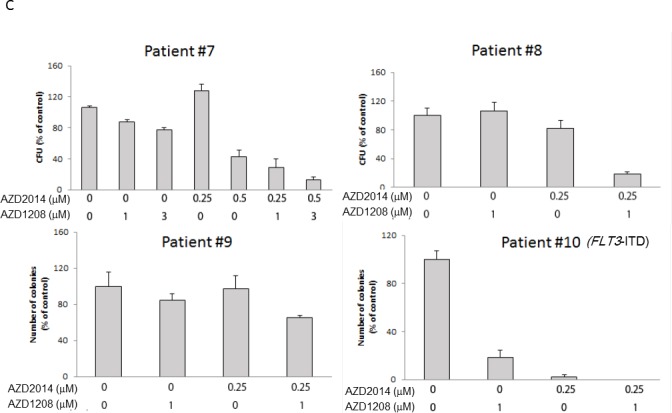
Synergistic interaction between AZD1208 and AZD2014 in AML cells **A.** MOLM-13, MOLM-14, MOLM-16, MV4;11, and OCI-AML3 AML cells were cultured for 72 h in the presence of escalating doses of AZD1208, AZD2014, or the combination at a fixed ratio (10:1, except MOLM-16, 4:1). The AZD1208 concentrations used were 0, 0.5, 1, or 2 μM for MOLM-16 and 0, 2.5, 5, or 10 μM for the other cell lines; the AZD2014 concentrations used were 0, 0.125, 0.25, or 0.5 μM for MOLM-16 and 0, 0.25, 0.5, or 1 μM for the other cell lines. The viable cells were counted by the trypan blue exclusion method and apoptosis was determined by Annexin V staining positivity. Graph shows the mean ± SEM of results of three independent experiments **B.** Cells derived from 6 primary AML samples were treated with AZD1208, AZD2014, or the combination in various concentrations in the presence of MSCs and tested for viability and cell death after 24 h by FACS. Patients #2-4 have *FLT3*-ITD mutation-positive disease. **C.** Clonogenic assay of cells derived from primary AML samples 14 days after AZD1208, AZD2014, or combination treatment. Patient #10 has *FLT3*-ITD mutation-positive disease.

To assess the effects of a combination of pan-PIM inhibitor AZD1208 and dual mTORC1/2 inhibitor AZD2014, we investigated the proliferation and survival of cultured AML cell lines treated with these agents. AML cells were treated with AZD1208, AZD2014, or their combination for 72 h, and the viable cells and Annexin V-positive cells were isolated and counted. As shown in Figure 2A, both AZD1208 and AZD2014 resulted in dose-dependent growth inhibition and cell death in AML cells (except MOLM-14, which were relatively resistant to AZD1208)*.* The sensitivity to PIM inhibitor AZD1208 varied among AML cell lines: MOLM-16 was extremely responsive to AZD1208, requiring the lowest concentration to induce cell growth inhibition and cell death, followed by OCI-AML3, MV4;11, and MOLM-13, which required progressively higher doses. To assess the interaction between PIM and mTOR inhibitors, we used Calcusyn software to analyze their effects on growth inhibition according to the Chou-Talalay method [20, 21]; this analysis demonstrated a very strong synergistic effect between AZD1208 and AZD2014 in MOLM-16, a strong synergistic effect in MV4;11, synergism in MOLM-14 and OCI-AML3, and a slight antagonistic effect in MOLM-13 cells (Table 1).

**Table 1:** Combination indices for AZD1208 and AZD2014 in AML cell lines

Combination of AZD1208 and AZD2014	MOLM16	MOLM14	MV4;11	MOLM13	OCI-AML3
ED50	<0.01	2.51	4.91	0.95	0.79
ED75	<0.01	1.25	0.97	0.46	0.54
ED90	<0.01	0.63	0.19	0.22	0.37
CI average	<0.01	0.36	0.25	1.2	0.31

ED; effective dose, CI; combination index

Next we tested the effect of AZD1208 and AZD2014 on primary AML samples with either *FLT3*-WT or *FLT3*-ITD status (Table S1) under stromal co-culture conditions that used bone marrow (BM)-derived mesenchymal stem cells (MSCs) to mimic physiologic conditions [22-24]. As shown in Figure 2B, AZD1208 induced moderate growth inhibition of cells from 2 of 6 primary AML samples (Patients #2 and #3) and AZD2014 had the same effect in 4 of 6 samples (Patients #1-#3 and #6). In particular, cells from Patient #2 were considerably more sensitive than cells from the other samples to both AZD2014 and AZD1208. Combination of AZD1208 and AZD2014 further reduced cell viability and increased the percentage of Annexin V-positive cells in all the AML samples except those from Patients #4 and #5, which responded only to AZD2014. The sensitivity to AZD1208 and AZD2014 in AML patient-derived cells seemed unaffected by *FLT3* mutation status. Similar trends were observed in the same experiments carried out without MSC co-culture (Figure S2). The responses of AML cell lines and primary AML samples to the PIM inhibitor indicate that the sensitivity to PIM inhibition is independent of *FLT3*-ITD status.

Importantly, the clonogenic assay revealed the complementary effect of the 2 drugs in all the primary sample-derived cells tested regardless of *FLT3* mutation status (Figure 2C and Table S2). Notably, additive effects were seen in samples from Patients #8 and #9, in which each inhibitor alone exhibited only moderate or no effect.

### Effects of AZD1208 and AZD2014 on downstream signaling pathways

**Figure 3:**
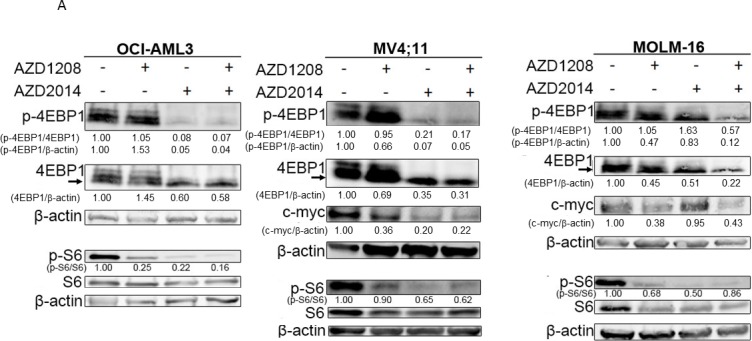

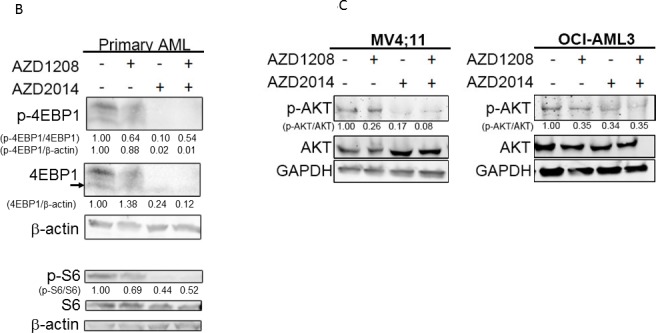

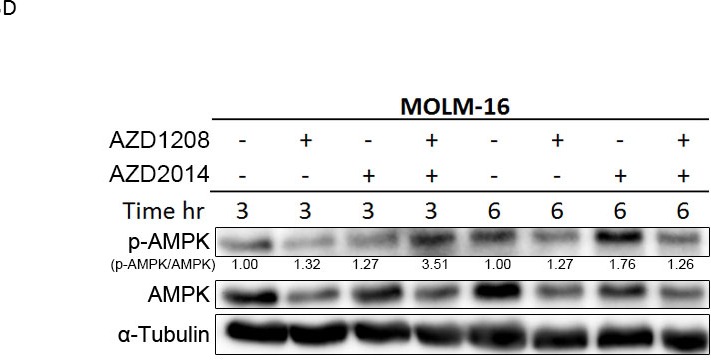

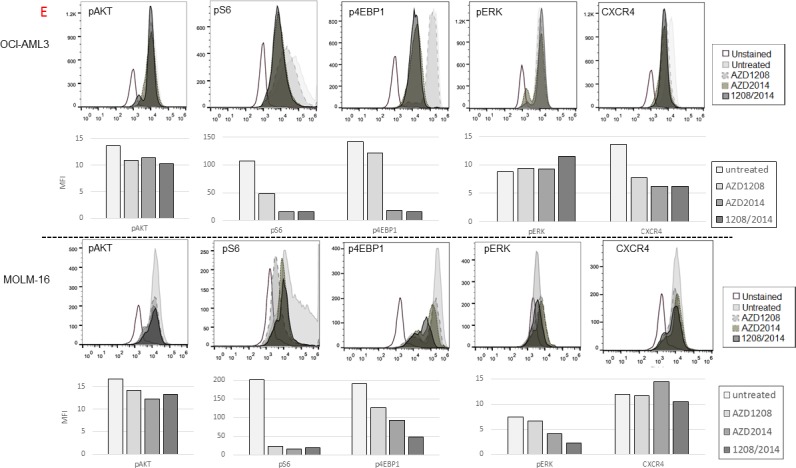
Molecular pathways affected by synergistic inhibition of AZD1208 and AZD2014 Treated AML cells were subjected to Western blotting to detect various proteins as indicated. **A.** Cells were incubated with 10 μM of AZD1208 (1 μM for MOLM-16), 1 μM of AZD2014, or the combination for 3 h (OCI-AML3 and MV4;11) or 6 h (MOLM-16). **B.** Cells derived from primary AML samples (BM with 60% blasts from Patient #6) were treated for 6 h with 3 μM of AZD1208, 1 μM of AZD2014, or the combination. **C.** Cells were incubated with 10 μM of AZD1208 (1 μM for MOLM-16), 1 μM of AZD2014, or the combination for 24 h. **D.** MOLM-16 cells were incubated with 3 μM of AZD1208, 1 μM of AZD2014, or the combination for indicated times. **E.** Measurement of the mean fluorescence intensities (MFI) of intracellular pAKT, pS6, pEBP1, pERK, and CXCR4 in OCI-AML3 and MOLM-16 cells after indicated treatments for 6 h. Concentrations used: 1 μM of AZD2014 and 3 μM of AZD1208 for OCI-AML3 and 1 μM of AZD2014 and 2 μM of AZD1208 for MOLM-16. The intensity of protein levels were obtained by Image J software. The intensity of total 4EBP1 was quantified by using the lower unphosphorylated band.

Having confirmed the synergistic effect of PIM inhibitor AZD1208 and mTOR inhibitor AZD2014, we next examined the molecular pathway of cell death induced by these inhibitors using Western blotting and flow cytometry. Eukaryotic initiation factor 4E-binding protein (4EBP1), one of the key molecules in mTOR pathway-mediated CAP-dependent translation, is the known downstream target of AZD1208 in AML [18, 25]. Phosphorylation of S6 at Ser240/244, another biomarker for mTOR activity, is the downstream target of mTORC1 and mTORC2. As expected, mTOR inhibitor AZD2014 and the combination of AZD1208 and AZD2014 rapidly reduced the phosphorylation level of 4EBP1 (Thr37/46) and S6 (Ser240/244) in cell lines OCI-AML3, MOLM-16, and MV4;11 (Figure 3A and 3E). Similar results were obtained in cells derived from the primary AML samples treated with AZD1208 and AZD2014 (Figure 3B). Treatment with the combination of AZD1208 and AZD2014 reduced the phosphorylation level of both 4EBP1 and S6 to a greater extent than either drug alone in MOLM-16. Reduction of phosphorylated p-4EBP1 detectable by phospho-specific antibody was also apparent by probing with total 4-EBP1 antibody, whereby mTOR inhibitor AZD2014 alone or combined with PIM kinase inhibitor AZD1208 caused disappearance of the upper, slower mobility phosphorylated bands of 4EBP1; moreover, some reduction of the lower unphosphorylated band was seen in all cell lines tested. c-Myc, another biomarker for the induction of leukemogenesis and frequently activated in AML, is known to work in concert with the PI3K/AKT/mTORC1 signal transduction pathway [26]. In *FLT3*-ITD cell line MV4;11, both AZD1208 and AZD2014 as single agents and in combination reduced c-Myc protein levels while in *FLT3*-WT cell line MOLM-16, only AZD1208 reduced c-Myc protein level (Figure 3A).

The association of AKT phosphorylation with cell survival has been reported previously [27]. AKT is upstream of mTORC1 and downstream of mTORC2 in the mTOR signaling pathway [27]. Like the other mTOR targets, AKT phosphorylation was reduced after treatment with AZD1208, AZD2014, or the combination in MV4;11 and OCI-AML3 cells (Figure 3C and 3E). Furthermore, phosphorylation of AMP-activated protein kinase alpha (AMPKα, Thr172) was rapidly induced after only 3 h of the combination treatment but not AZD1208 or AZD2014 alone (Figure 3D). AZD2014, AZD1208, and their combination reduced CXCR4, a known mediator of AKT activation [28], in OCI-AML3 but not MOLM-16 cells, in which the AZD1208 and AZD2014 combination reduced phosphorylation of ERK (Figure 3E).

### Proteomic profiling of combined AZD1208 and AZD2014 treatment in AML cells

To investigate the pathways altered by AZD1208 and AZD2014, either as monotherapy or in combination, we employed a proteomic approach using isobaric tags for relative and absolute quantitation (iTRAQ) analysis, which measures mature proteins as an approach to closely examining possible biological changes in cells. As shown in Table 2, the iTRAQ analysis revealed that AZD1208, AZD2014, and their combination caused changes in various proteins. We extracted lists of proteins commonly altered by these drugs both in MOLM-16 and OCI-AML3 cells and found that AZD1208 reduced elongation factor 1-alpha 1 (EF1A1) while AZD2014 reduced elongation factor 2 (EF2), revealing that the translation elongation process is negatively regulated by these agents (Table 3). Interestingly, the combination treatment inhibited these targets to a greater extent than monotherapy; moreover, the combination therapy reduced other protein synthesis-related factors, including elongation factors such as eukaryotic initiation factor 4a-1 (IF4a1) and ribosomal protein S4, X-linked (RS4X), whereas it induced proapoptotic gene *CH10* (HSPE1). Metabolic pathway-related proteins such as fatty acid synthase also were significantly downregulated by the combination (Table 3).

**Table 2:** Numbers of proteins whose expression was altered by AZD1208, AZD2014, or their combination in AML cells*

	AZD1208		AZD2014		Combination	
	up	down	up	down	up	down
OCI-AML3	3	11	14	30	26	31
MOLM-16	41	28	19	15	72	82

* Detected by iTRAQ analysis

up, upregulated; down, downregulated

**Table 3:** Frequently altered proteins in OCI-AML3 and MOLM-16 cells after treatment with AZD1208, AZD2014, or the combination*

AZD1208			
GeneSymbol	Protein name	Fold changes	
		OCI-AML3	MOLM16
*ALBU*	Serum albumin	1.8762	1.3757
*RPN1*	ribophorin I	0.9292	1.1966
*HSP7C*	Heat shock cognate 71 kDa protein	0.9089	0.7391
*EF1A1*	Elongation factor 1-alpha 1	0.8668	0.7897
AZD2014			
GeneSymbol	Protein name	Fold changes	
		OCI-AML3	MOLM16
*1-/S90A*	Isoform 2 of Heat shock protein HSP 90-alpha	0.9327	0.9098
*EF2*	eukaryotic translation elongation factor 2	0.8673	0.9314
*DNJA1*	DnaJ homolog subfamily A member 1	0.7815	0.7554
*NP1L1*	Nucleosome assembly protein 1-like 1	0.857	0.7198
AZD1208/AZD2014 combination			
GeneSymbol	Protein name	Fold changes	
		OCI-AML3	MOLM16
*ALBU*	Serum albumin	2.434	1.8055
*RPN1*	ribophorin I	0.912	1.1888
*HMGS f*	high mobility group box 1	1.1893	1.1948
*HSP7C*	Heat shock cognate 71 kDa protein	0.8319	0.8472
*EF2*	Elongation factor 2	0.8615	0.8857
*GLU2B*	Glucosidase 2 subunit beta	1.1713	1.319
*HMGB2*	High mobility group protein B2	1.1918	1.2797
*DNJA1*	DnaJ homolog subfamily A member 1	0.7526	0.6752
*PHB*	prohibitin	1.1208	1.8256
*MDHM*	Malate dehydrogenase, mitochondrial	1.1328	1.2432
*FAS*	Fatty acid synthase	0.859	0.8684
*ACLY*	ATP citrate lyase	0.9216	0.8802
*NP1L 1*	Nucleosome assembly protein 1-like 1	0.8495	0.7276
*1F4A1*	Eukaryotic initiation factor 4A-I	0.841	0.7729
*CH10*	10 kDa heat shock protein, mitochondrial	1.1281	1.2642
*RS4X*	40S ribosomal protein S4, X isoform	0.888	0.8417
*H4*	histone cluster 1, H4f	2.0811	2.8924
*EF1A1*	Elongation factor 1-alpha 1	0.7936	0.8127
*RL24*	60S ribosomal protein L24	1.3691	0.8494
*HS105*	Heat shock protein 105 kDa	0.8455	0.8865

* Detected by iTRAQ. Expression of all proteins listed was significantly different (*P* < 0.05) between controls and cells treated with AZD1208, AZD2014, or the combination. Values indicate fold-changes relative to untreated cells. Confidence score (a percentage measure of the confidence of the protein identification) for all proteins in the table was 99%.

Using DAVID Bioinformatics Resources [29, 30] and the Ingenuity Pathway Analysis (IPA) bioinformatics tool [31], we performed pathway analysis to characterize the drug-induced alterations in biological processes (Table 4). In AZD1208/AZD2014-sensitive MOLM-16 cells, the pathway most altered by AZD1208 was the ribosome biogenesis pathway, while the pathway most altered by AZD2014 was the glycolysis/gluconeogenesis pathway. Combined, these inhibitors altered ribosome biogenesis, as expected (Table 4). In OCI-AML3 cells, which were moderately responsive to either AZD1208 or AZD2014, only the combination of AZD1208 and AZD2014 altered the ribosome pathway, suggesting that the combination of AZD1208 and AZD2014 might enhance functional alteration of protein synthesis (Table 4).

**Table 4:** Gene ontology of altered genes in OCI-AML3 and MOLM-16 cells after treatment with AZD1208, AZD2014, or the AZD1208/AZD2014 combination*

**MOLM16**		
AZD1208		
Gene ontology of altered genes	Altered gene count	P Value
Ribosome	7	3.E-05
Alzheimer's disease	8	1.E-04
Parkinson's disease	7	3.E-04
Huntington's disease	7	2.E-03
Oxidative phosphorylation	6	2.E-03
Antigen processing and presentation	5	3.E-03
		
AZD2014		
Gene ontology of altered genes	Altered gene count	P Value
Glycolysis / Gluconeogenesis	5	4.E-05
		
AZD1208/AZD2014		
Gene ontology of altered genes	Altered gene count	P Value
Ribosome	33	6.E-36
Parkinson's disease	11	7.E-05
Huntington's disease	11	1.E-03
Oxidative phosphorylation	9	2.E-03
Alzheimers disease	9	8.E-03
Glycolysis / Gluconeogenesis	5	2.E-02
		
**OCI-AML3**		
AZD1208		
Gene ontology of altered genes	Altered gene count	P Value
Pentose phosphate pathway	2	3.E-02
		
AZD2014		
Gene ontology of altered genes	Altered gene count	P Value
Huntington's disease	6	1.E-03
Parkinson's disease	5	2.E-03
		
AZD1208/AZD2014		
Gene ontology of altered genes	Altered gene count	P Value
Spliceosome	5	5.E-03
Ribosome	4	1.E-02
Systemic lupus erythematosus	4	2.E-02
Synthesis and degradation of ketone bodies	2	5.E-02

* Determined by KEGG pathway enrichment analysis.

Each KEGG pathway enrichment analysis contains the indicated number of altered gene count and enrichment P values.

The IPA platform highlighted repression of the transcription factor heat shock factor 1 (HSF1), a central upstream regulator, by the combination of AZD1208 and AZD2014 in both the MOLM-16 and OCI-AML3 cell lines (Figure S3). Previous reports suggest that HSF1 activity is reflected by a range of post-translational modifications [32, 33], which might explain the lack of detectable change in total HSF1 protein level in the analysis. HSF1 is a master regulator of various cellular functions, including proliferation, survival, ribosome biogenesis, and glucose metabolism [34, 35], and thus suppression of the HSF1 pathway indicates efficient repression of ribosome biogenesis, leading to cell cycle inhibition. In both cell lines, upstream regulator Myc was downregulated by AZD1208 and Myb was downregulated by AZD2014. The combination caused downregulation of both Myc and Myb, emphasizing its synergistic effect on two oncogenic transcription factors playing critical roles in cellular proliferation and apoptosis.

### Translational repression by AZD1208 and AZD2104

**Figure 4:**
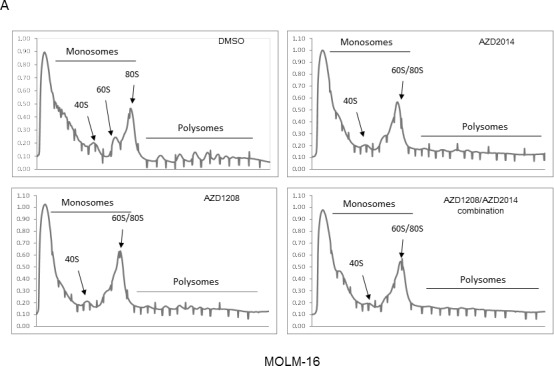

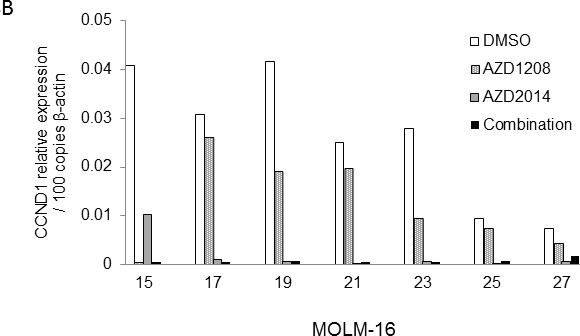
Polysome profiles of MOLM16 AML cells treated with AZD1208 and/or AZD2014 **A.** (i) Representative polysome profiles identified by velocity separation of translation complexes in linear sucrose gradients of MOLM-16 cells treated with DMSO (control), 2 μM AZD1208, 1 μM AZD2014, or the combination. (ii) The area under the curve for MOLM-16 cells was quantified by Image J software. **B.** qRT-PCR analysis of *CCND1* mRNA associated with polysomal fractions 15-27 in MOLM-16, normalized against DMSO treatment as indicated.

The PI3K/AKT/mTOR signaling pathway plays an important role in polysome formation, which controls CAP-dependent translation for protein synthesis [36]. We therefore performed polysome fractionation analysis in MOLM-16 and OCI-AML3 cells treated with AZD1208, AZD2014, or the combination to test whether the polysome fraction was altered by either of the drugs. We found that AZD1208 as well as AZD2014 altered the polysomal profile, indicating a reduced level of CAP-dependent translation (Figure 4A).

We next focused on the changes induced by these drugs in the polysome association of gene transcripts, which indicates the mode of translation. We assessed the polysome association of known CAP-dependent translation target cyclin D1, which is overexpressed and often associated with chemotherapeutic resistance in AML [37]. Figure 4B illustrates the levels of *cyclin D1 (CCND1)* transcripts present in each sucrose gradient fraction relative to *β-actin* in MOLM-16 cells. In these cells, AZD2014 alone or in combination with AZD1208 strongly reduced the polysome association of *CCND1* transcripts,** whereas AZD1208 alone had weak influence on the *CCND1*-polysome association (Figure 4B).

## DISCUSSION

In this study, we used *in vitro* screening of a novel drug combination, pan-PIM inhibitor AZD1208 and dual-mTORC1/2 inhibitor AZD2014 in AML. Our findings demonstrate that these inhibitors when used in combination arrest protein synthesis through simultaneous inhibition of the mTORC1/2 pathway and induce apoptosis in AML cells.

While Oncomine data analysis indicated that *FLT3* mutations are associated with high levels of PIM1 expression in AML patients, our analysis demonstrated that *FLT3*-ITD mutation status was not associated with differential levels of PIM1, 2, and 3 protein expression and did not confer higher sensitivity to PIM inhibition. Importantly, a clonogenic assay revealed the complementary effect of the 2 drugs in all the primary AML cells tested regardless of *FLT3*-ITD mutation status, suggesting the efficacy of prolonged treatment with the combination at the level of AML progenitor cells. Furthermore, considering the proto-oncogenic property of PIM kinases, our results showing that combined AZD2014 and AZD1208 had various levels of synergy, stronger in *FLT3*-WT cell lines than in *FLT3*-ITD mutant cells lines, indicate that *FLT3*-ITD-overexpressing mutation might affect pathways others than PIM and mTOR downstream signaling and thus escape the inhibitory effect of the combination of AZD1208 and AZD2014.

Consistently with previous reports [7, 25, 38], AZD2014 inhibited the mTORC1 pathway, as was shown by reduced phosphorylation of phospho-(p-)S6K (Ser-240-244) and p-4EBP1 (Thr-37/46), as well as the mTORC2 pathway, as indicated by reduced phosphorylation of p-AKT (Ser473). PIM kinases phosphorylate TSC2 to activate mTORC1 and phosphorylate 4EBP1 with activation of eIF4E, resulting in CAP-dependent translation [39]. Although the combined treatment with AZD1208 and AZD2014 caused a relative decrease of the p-4EBP1/total 4EBP1 (the lower unphosphorylated band) ratios, the amount of unphosphorylated 4EBP1 bands were also decreased in all tested cell lines and in a primary AML sample. This paradox can be explained by the finding that reduced eIF4E activity by hypophosphorylated 4EBP1 leads to degradation of its repressor protein 4EBP1 through ubiquitination [40]. AMPK is a known negative regulator of mTORC1 [41], and the pan-Pim kinase inhibitor or PIM1 siRNA has been shown to block mTORC1 activity by stimulating the phosphorylation and thus activation of AMPK [42]. Concordant with these reports, our results indicate the contribution of the AMPK-mTOR signaling pathway to the greater antitumor effects of the AZD1208 and AZD2014 combination than either drug alone. Moreover, the AZD1208 and AZD2014 combination reduced CXCR4 surface expression or ERK phosphorylation in a cell line-dependent manner. CXCR4 is a known mediator of AKT and ERK activation [28, 43] as well as a target of PIM [44], and several studies demonstrated the downregulation of p-ERK by PIM1 knockdown or by treatment with a PIM1 inhibitor [45, 46]. Taken together, our findings indicate the possible antitumor effects of simultaneous inhibition of mTOR and PIM through depletion of the mTORC1/2 feedback reaction and repression of known and putative PIM1 kinase downstream targets.

Our protein expression analysis and profiling of relevant pathways revealed suppression of Myc by the mTOR inhibitor, of Myb by the PIM inhibitor, and of HSF1 by either. Although the *Myc* oncogene is known to directly regulate the protein synthesis machinery, recent reports suggest that, in *Myc*-driven cancers such as AML, Myc enhances protein synthesis by activating mTOR-dependent phosphorylation of 4EBP1 [47]. Both Myc and Myb, another oncogenic transcription factor, might play roles in the antitumor efficacy of the AZD1208 and AZD2014 combination [48]. Notably, HSF1 inhibition was frequent in OCI-AML3 and MOLM-16 cells treated with AZD1208, AZD2014, or the combination. In cancer cells, translation is driven by HSF1 through regulation of ribosome biogenesis [49, 50], and HSF1-dependent transactivation is regulated by mTOR activity [51]. Global screening by iTRAQ analysis, which detected the small but significant (*P* < 0.05) differences in expression of multiple proteins in the same pathway [47], indicates that the combination of AZD1208 and AZD2014 directs cells to mitochondria-mediated apoptosis through mTOR inhibition. Fatty acid synthase, one of the proteins most significantly downregulated by the combination regimen (but not by either drug alone), is a possible downstream target of the PI3K/AKT/mTOR pathway [52]. Inhibition of fatty acid synthesis is known to inactivate AKT and promote apoptosis in bladder transitional cell carcinoma [53]. We have further demonstrated impaired polysome formation in AML cells treated with the mTOR inhibitor and PIM inhibitor and reduction of polysome formation of the oncoprotein cyclinD1, possibly through suppression of the HSF pathway. Finally, proapoptotic protein CH10 (HSPE1) was induced only by the combination [54]. While our analyses demonstrated several downstream targets modulated by the combination, it is conceivable that not a single target, but multiple downstream effectors, contribute to the observed growth-inhibitory effects. Recently, Meja *et al*. [55] demonstrated the synergistic cytotoxicity of a combination of AKT and PIM inhibitors in AML, with convergence on inhibition of Myc, MCL-1, and BAD. It is well documented that mTOR signaling contributes to multiple aspects of cancerous behavior, including metastasis, survival, and chemotherapy resistance [56, 57]. Importantly, AZD2014 is now at the stage of a phase II clinical trial for solid tumors after the safety and tolerability were documented in a phase I trial [58].

In summary, our findings provide the first evidence that the combination of dual mTOR inhibitor AZD2014 and pan-PIM inhibitor AZD1028 effectively reduces protein synthesis by simultaneous inhibition of the mTORC1/2 pathway and induces apoptosis in AML cells. These findings suggest that this combination is an attractive therapeutic strategy for AML and warrants further clinical investigation.

## MATERIALS AND METHODS

### Cells and culture conditions

The following *FLT3*-ITD mutant AML cell lines were used: MV4;11 [59], purchased from American Type Culture Collection (Manassas, VA); MOLM-13 [60], provided by Hayashibara Biochemical Laboratories (Okayama, Japan); and MOLM-14 [60], provided by Dr. Mark Levis (Johns Hopkins University School of Medicine, Baltimore, MD). *FLT3*-WT AML cell lines OCI-AML3 [61] and MOLM-16 were purchased from DSMZ (Braunschweig, Germany). Cells were cultured in RPMI 1640 medium containing 10% fetal bovine serum (FBS), 1% L-glutamine, and 1% penicillin-streptomycin at 37°C in 5% CO_2_.

To represent the BM stroma, MSCs obtained from healthy BM donors and AML patients were cultured at a density of 5,000 to 6,000 cells/cm^2^ in minimum essential medium alpha supplemented with 20% FBS, 1% L-glutamine, and 1% penicillin-streptomycin as described elsewhere [62]. Passage 3 or 4 MSCs were used for the co-culture experiments. To study the effect of BM stroma on AML cells, MOLM-13 and HL60 cells were cultured at a density of 5×10^5^, with or without a layer of MSCs plated at a density of 0.2×10^5^ cells/cm^2^. Co-cultured AML cells were separated from the MSC monolayer by careful pipetting with ice-cold phosphate-buffered saline solution (PBS), repeated twice. After the AML cells were collected, to rule out the possibility of contamination with MSCs, MSC monolayers were examined by microscopy (×100) to confirm that the monolayer was not damaged and that fewer than 10 leukemic cells per visual field remained attached. To verify lack of significant contamination in collected leukemic cells, they were analyzed by flow cytometry using CD45 as a discriminator between leukemic cells and MSCs [62]. Data were acquired and analyzed by using CellQuest software (Becton Dickinson Immunocytometry Systems, San Jose, CA). In indicated experiments, co-cultures were performed in the presence of the pan-PIM inhibitor AZD1208 and/or the dual mTOR inhibitor AZD2014; both were provided by AstraZeneca R&D (Waltham, MA).

Peripheral blood and BM samples were obtained from patients with AML after informed consent was obtained in accordance with a protocol approved by The University of Texas MD Anderson Cancer Center Institutional Review Board regulations. Ficoll-Hypaque density gradient centrifugation was used to separate mononuclear cells (Sigma-Aldrich, St Louis, MO).

### Cell viability and apoptosis assays

Cell viability, apoptosis, and specific cell death were assessed by the trypan blue exclusion cell count method and by Annexin V/7AAD staining positivity analyzed by a fluorescence-activated cell sorting FACS Array Bioanalyzer (BD Biosciences, San Jose, CA) as described previously [63].

### Flow cytometry for detection of the expression level of intracellular phospho-proteins

MOLM-16 and OCI-AML3 cells were treated for 6 h with AZD1208 (2 μM), AZD2014 (1 μM), or the combination. Cells were then fixed with 1.6% paraformaldehyde (Electron Microscopy Sciences, Hatfield, PA) and subjected to permeabilization in ice-cold methanol (70% in PBS; 1 mL/million cells) for 20 min. After washing twice, cells were resuspended in 1% bovine serum albumin in PBS. Antibodies were added to the cell suspension and incubated for 30 min. Antibodies used were Phospho-p44/42 MAPK (Erk1/2) (Thr202/Tyr204) (E10) mouse monoclonal antibody (Alexa Fluor 488 Conjugate #4374; Cell Signaling Technology, Beverly, MA); Phospho-S6 Ribosomal Protein (Ser235/236) (D57.2.2E) XP rabbit monoclonal antibody (Alexa Fluor 647 Conjugate #4851; Cell Signaling Technology); Phospho-Akt (Ser473) rabbit monoclonal antibody (Alexa Fluor 647 Conjugate #A88881; Beckman Coulter, Inc., Brea, CA); and CXCR4 (CD184-APC, #555976; BD Biosciences). After washing twice, cells were resuspended and analyzed by a Gallios flow cytometer (Beckman-Coulter).

### Quantitative real-time PCR

RNA (50-100 ng) from polysomal fractions isolated by using the RNeasy kit (QIAGEN, Valencia, CA) was used to synthesize cDNA with the iScript Reverse Transcription Supermix (Bio-Rad Laboratories, Hercules, CA). Real-time polymerase chain reactions (PCR) were then performed on a 7900 System (Applied Biosystems, Grand Island, NY) with a SYBR Green qPCR kit (Applied Biosystems) and gene-specific primers for β-actin (ACTB VHPS110; RealTimePrimers.com) and cyclin D1 (CCND1; Hs.PT.56a.4930170; IDT).

### Clonogenic assay

Mononuclear cells were seeded at 0.05-0.1×10^6^ cells/mL in Methocult H4435 (Stem Cell Technologies, Vancouver, BC, Canada). AZD1208 (1 or 3 μM), AZD2014 (0.25 or 0.5 μM), or the combination was added to the medium before plating. The cells were subjected to vortexing for 15 s and were then plated on 35×10-mm dishes with a 2×2-mm grid (NUNC) in triplicate and incubated in a humidified chamber at 37°C in 5% CO_2_ for 14 days. Colonies were scored by using a 1×-3 stereoscope (VWR, Radnor, PA).

### Western blot analysis

Cells were solubilized in lysis buffer (i.e., PBS containing 1× cell lysis buffer [Cell Signaling Technology], 1× protease inhibitor cocktail [Roche, Indianapolis, IN], and 1× phosphatase inhibitor cocktail I/II [Calbiochem, San Diego, CA]) and incubated for 30 min on ice. The lysates were then subjected to centrifugation for 10 min at 13,000 rpm at 4°C. Protein concentration was determined by the Bio-Rad Protein Assay Kit (Bio-Rad Laboratories) according to the manufacturer's instructions. Total proteins (40 μg) were separated by sodium dodecyl sulfate-polyacrylamide gel electrophoresis (Bio-Rad Laboratories) and transferred to polyvinylidene-fluoride membranes (0.45 μm, GE Healthcare, Buckinghamshire, UK), then probed with first and second antibodies according to the manufacturers’ protocols. For immunoblotting, the following antibodies were used: alpha-tubulin and beta-actin (Sigma-Aldrich), PIM1 (Abcam, Cambridge, MA), PIM3 (Santa Cruz Biotechnology, Santa Cruz, CA), PIM2, p70 S6 Kinase, phospho-(p-) p70 S6 Kinase (Thr389), 4EBP1, phospho-(p-)4EBP1 (Thr37/46), AKT, p-AKT (Ser473), AMPK-α, pAMPK-α (Thr172), c-myc, GAPDH, and horseradish peroxidase-linked anti-mouse and anti-rabbit IgG (all from Cell Signaling Technology).

iTRAQ sample labeling, mass spectrometry analysis, and peptide identification

The iTRAQ chemical labeling mass spectrometry (MS) method was performed by following the manufacturer's protocol [64]. Briefly, cell lysates were subjected to liquid chromatography (LC)-shotgun analyses using the iTRAQ method as described previously [65]. Prior to iTRAQ analysis, lysates were concentrated and buffer exchanged by using 3.5-kDa molecular weight cut-off spin concentrators (Tomy Seiko Co., Ltd, Tokyo, Japan), then digested for 24 h with 10 μg trypsin treated with L-1-(4-tosylamido)-2-phenylethyl tosylphenylalanyl chloromethyl ketone. Each peptide solution was labeled with one of the 4 iTRAQ reagents (iTRAQ reporter ions of 114, 115, 116, or 117 mass/charge ratios) according to the manufacturer's protocol (AB SCIEX, Framingham, MA). The tag labeling order was 114 for controls, 115 for AZD1208-, 116 for AZD2014-, and 117 for AZD1208/AZD2014 combination-treated cells. Labeled peptides were pooled and fractionated by strong cation exchange, using a ChromXP C18-CL column (Eksigent division of AB SCIEX, Dublin, CA), and analyzed by nano LC-MS/MS; nano LC-MS was performed on a TripleTOF 5600 mass spectrometer for MS/MS (AB SCIEX) interfaced with a nanoLC system (AB SCIEX) [66]. Proteins were identified and quantified relatively by applying ProteinPilot Software Version 4.5 (AB SCIEX) to the data as previously described [67]. Functional definitions of the protein contents were searched against the Swissport database (Release 10/16/2013) using the search algorithm within ProteinPilot Software and Analyst TF Software (AB SCIEX). Protein ratios were normalized by using the overall median ratio for all the peptides in the sample for each separate ratio in every individual experiment. A confidence cutoff for protein identification of >95% was applied. Proteins whose expression was statistically significantly changed by treatment were subjected to functional analysis by KEGG pathway enrichment analysis performed using DAVID Bioinformatics Resources or the Ingenuity Pathway Analysis software (Ingenuity Systems, QIAGEN; www.qiagen.com/ingenuity).

### Polysomal assay

Molm-16 and OCI-AML3 cells were treated for 6 h with 2 or 3 μM AZD1208, respectively, 1 μM AZD2014, or the combination. Cells were then washed twice with PBS supplemented with cycloheximide (100 μg/mL; Sigma) and resuspended in hypotonic lysis buffer (5 mM Tris, pH 7.5; 2.5 mM MgCl_2_; 1.5 mM KCl) supplemented with cycloheximide (100 μg/mL), dithiothreitol (2 mM, Sigma), protease inhibitor (Cocktail set V EDTA free; Calbiochem), and RNase inhibitor (1 U/μL; Life Technology). After the suspension was subjected to vortexing for 4 s, Triton ×100 (0.5%; Sigma) and sodium deoxycholate (0.5%; Sigma) were added to the mix. After spinning at 12,000*g* for 5 min at 4°C, the supernatant was transferred to a new tube and snap-frozen in liquid nitrogen. Polysomal fractionation was carried out as previously described [68].

### Statistical analysis

Synergism, additive effects, and antagonism were assessed by the Chou-Talalay method [21], utilizing Calcusyn software (Biosoft, Cambridge, UK). Cells were treated by serially diluted AZD1208 and / or AZD2014 with a ﬁxed constant ratio (4:1 for MOLM16, 10:1 for MOLM14, MV4;11. MOLM13, OCI-AML3). After 72 hours treatment, viable cell numbers were determined by the trypan blue exclusion cell count method. The effect on cellular proliferation was shown as a percentage reduction of cell viability when compared with dimethyl sulfoxide (DMSO)-treated controls. The mean of three independent experiments was obtained. The average combination index (CI) value for the experimental combination was calculated from the 50%, 75%, and 90% effective doses. By this method, CI values indicate the following: 0.3-0.7, strong synergism; 0.7-0.85, moderate synergism; 0.85-0.9, slight synergism; 0.9-1.1, nearly additive; 1.1-1.2, slight antagonism; 1.2-1.45, moderate antagonism; 1.45-3.3, antagonism; 3.3-10, strong antagonism [21].

## SUPPLEMENTARY MATERIAL TABLES AND FIGURES


