# Supplementary material for: The novel combination of dual mTOR inhibitor AZD2014 and pan-PIM inhibitor AZD1208 inhibits growth in acute myeloid leukemia via HSF pathway suppression
Source: Oncotarget. 2015 Oct 14;6(35):37930–47. doi: 10.18632/oncotarget.6122 (PMC4741975; doi:10.18632/oncotarget.6122)
Supplement: Supplementary file 1 [file oncotarget-06-37930-s001.pdf]

The novel combination of dual mTOR inhibitor AZD2014 and pan-PIM inhibitor AZD1208 inhibits growth in acute myeloid leukemia via HSF pathway suppression

Supplementary Material

Table S1. Blast count and FLT3-ITD status of AML patients whose samples were used for cytotoxic assay*

Patient #	FLT3 status	% Blasts
1	WT	99
2	ITD	92
3	ITD	40
4	ITD	44
5	WT	75
6	WT	60

*Results of cytotoxic assay shown in Figure 2B.
WT, wild type; ITD, internal tandem duplication mutation
Blast count and *FLT3*-ITD status of AML patients used for cytotoxic assay shown in Figure 2B.

Table S2. Clinical characteristics of AML patients whose samples were used for clonogenic assay*

Patient #	Age	% Blasts	Mutation Status
7	74	62	FLT3-wt
8	57	95	FLT3-wt
9	68	25	ASXL1, EZH2, TET2, NRAS, IDH2-multiple methylation and splice mutation pattern
10	67	83	FLT3-ITD (ratio >0.5, high expression)

* Clinical characteristics of AML patients used for clonogenic assay shown in Figure 2C.
wt, wild type; ITD, internal tandem duplication mutation

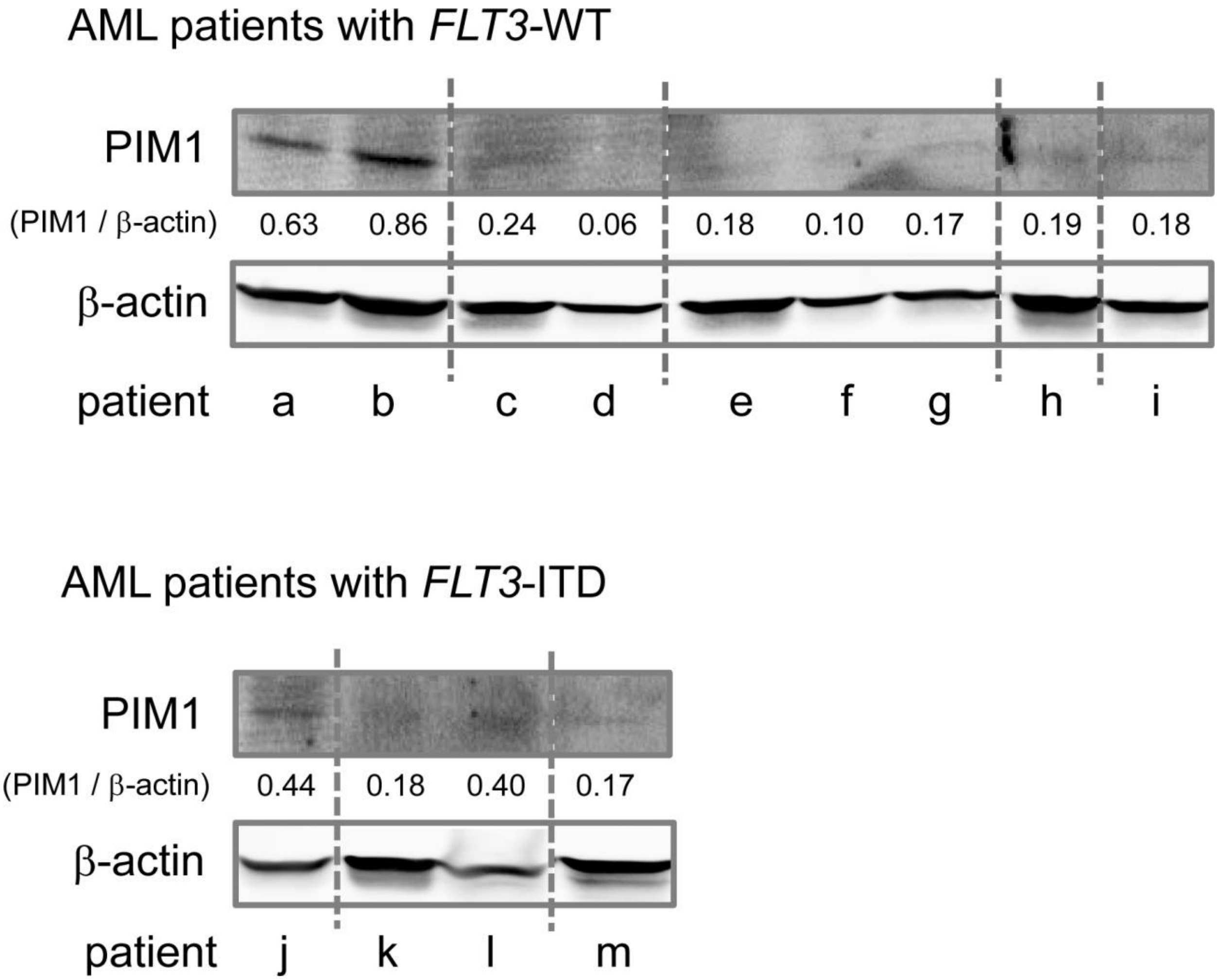

Figure S1. PIM1 protein levels in samples from AML patients with *FLT3*-WT (patient a – i) or with *FLT3*-ITD (patient j – m) were detected by Western blotting. The intensities compared to those of β−actin levels after background subtraction were quantified by Image J software.

Patient #1

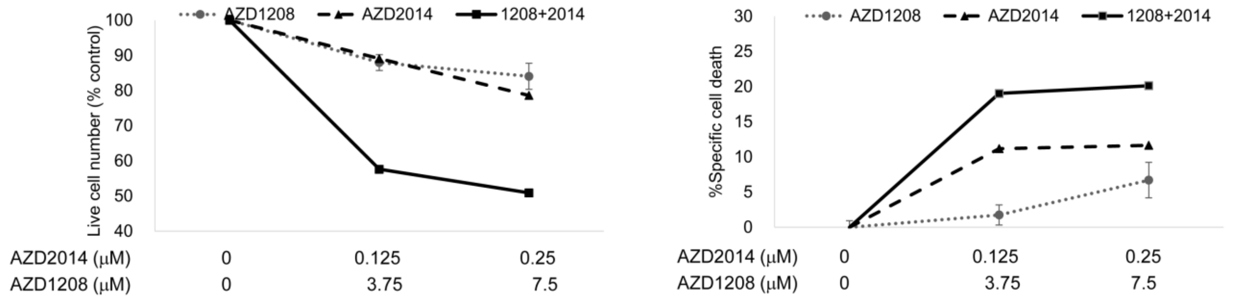

Patient #2 (*FLT3*-ITD)

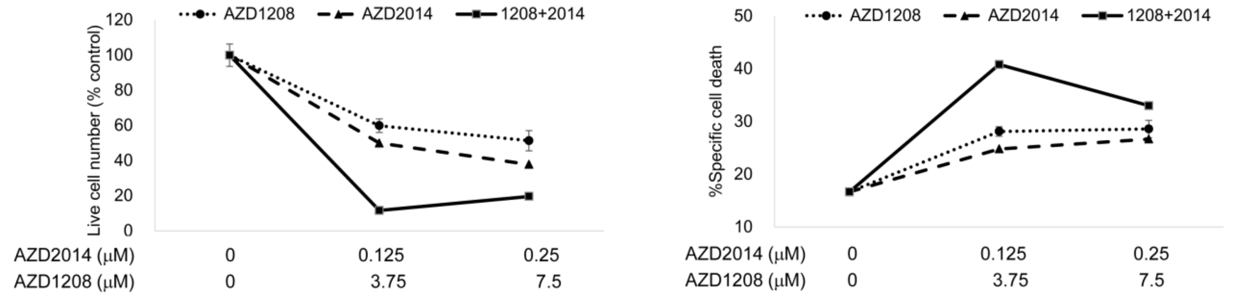

Patient #3 (*FLT3*-ITD)

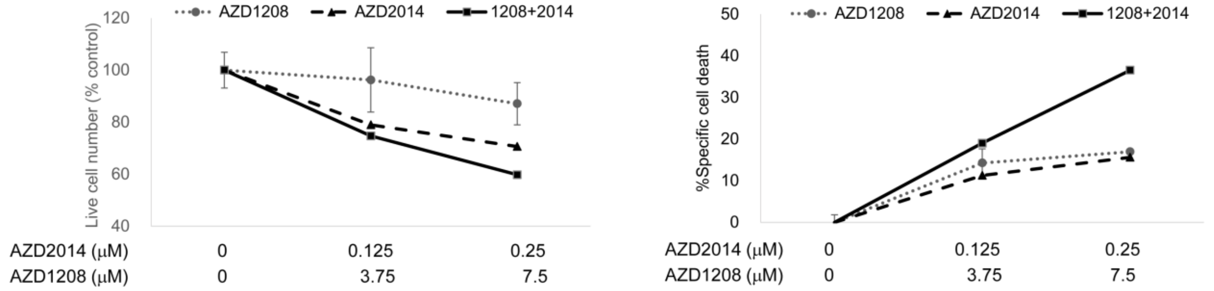

Patient #4 (*FLT3*-ITD)

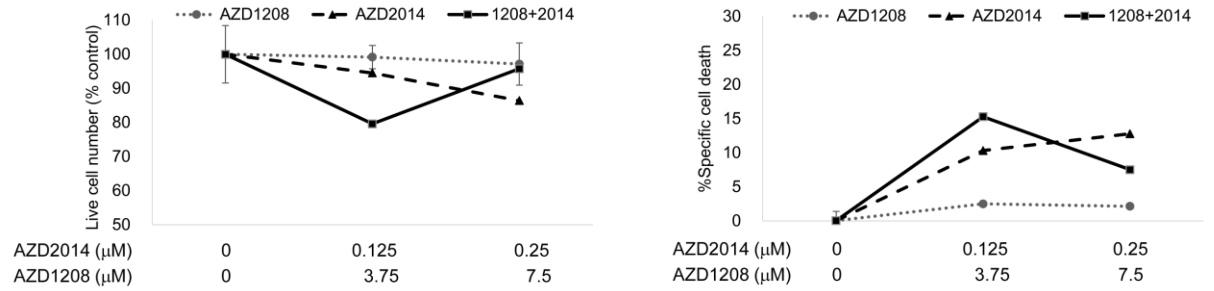

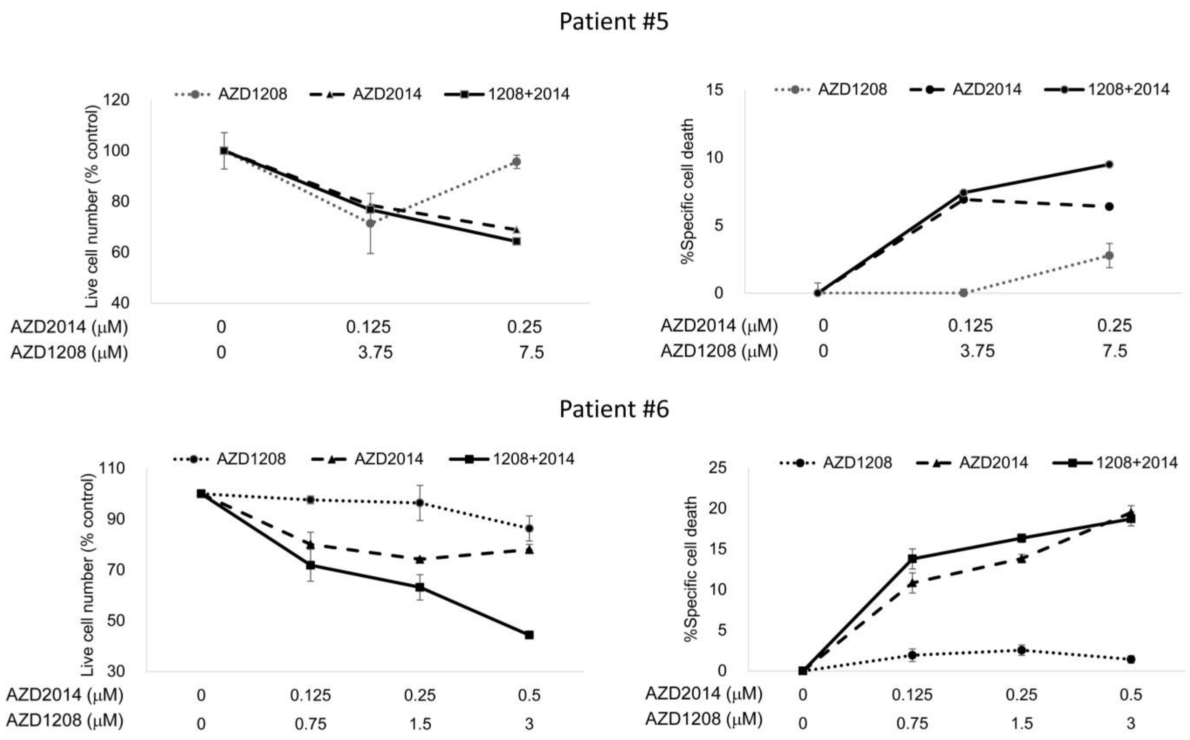

Figure S2. Primary AML cells from 6 patients were treated with AZD1208, AZD2014, or the combination in various concentrations in the absence of MSCs and tested for viability and cell death by Annexin V/7AAD flow cytometry after 24 h. Patients #2-#4 have *FLT3*-ITD mutation–positive disease.

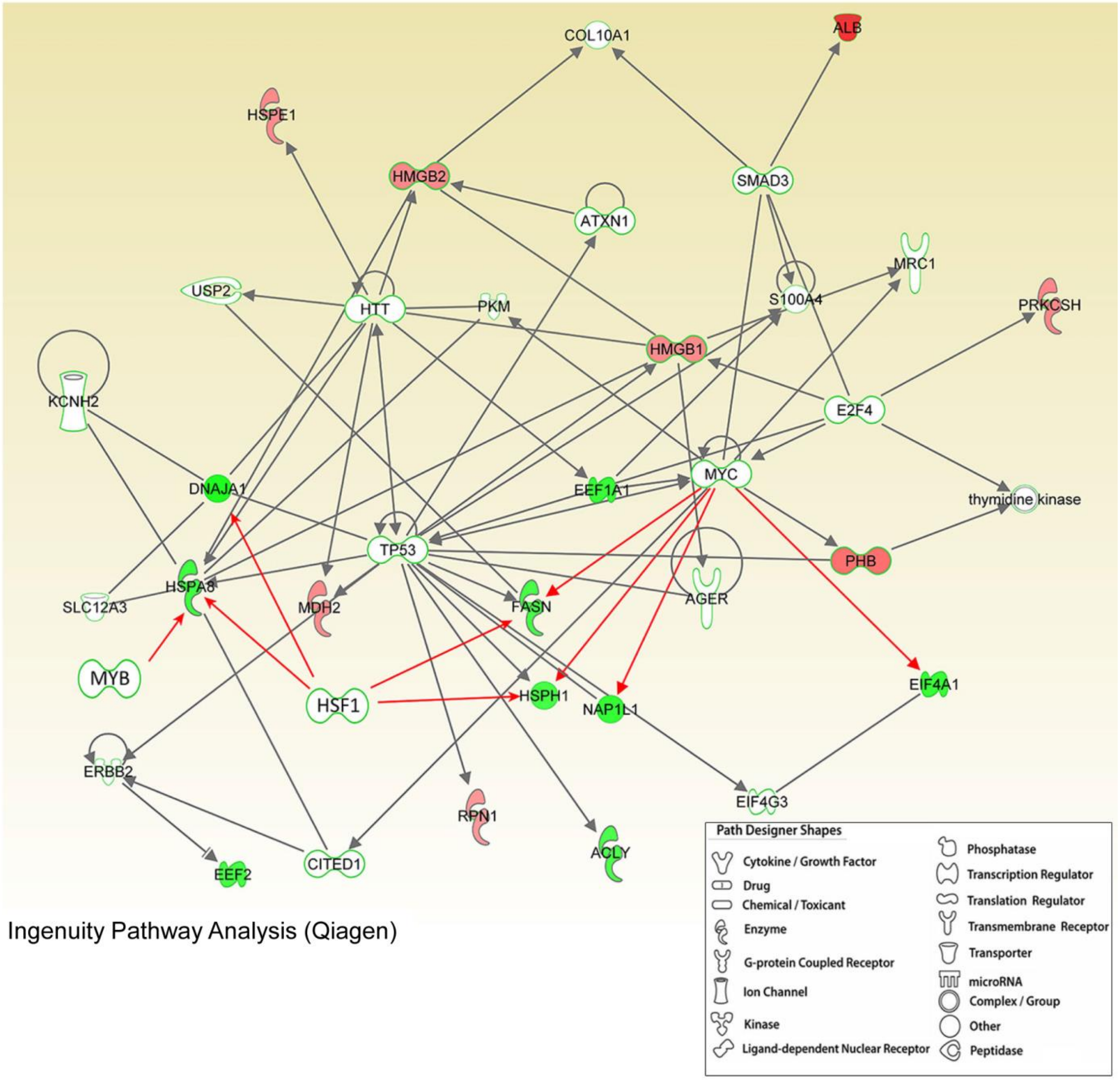

Figure S3. Network of proteins involved in AML responses to AZD1208 and AZD2104

Data were analyzed by Ingenuity Pathway Analysis (QIAGEN, www.qiagen.com/ingenuity). The IPA network analysis showing direct interactions between differentially expressed molecules in OCI-AML3 and MOLM-16 cell lines after combination treatment with AZD1208 and AZD2014. Arrows indicate direct interactions between molecules. The network with the highest score is shown. Upregulated proteins in the dataset are depicted in red and downregulated proteins in green. Red arrows indicate positive regulation.
